# Editorial: Advanced neuroimage techniques for metabolic and blood flow assessment in cerebrovascular disease

**DOI:** 10.3389/fneur.2023.1238378

**Published:** 2023-07-06

**Authors:** Wei Jin, Binyin Li, Yuan Feng, Yanmei Tie

**Affiliations:** ^1^Department of Neurology and Institute of Neurology, Ruijin Hospital, Shanghai Jiao Tong University School of Medicine, Shanghai, China; ^2^Clinical Neuroscience Center, Ruijin Hospital, Shanghai Jiao Tong University School of Medicine, Shanghai, China; ^3^School of Biomedical Engineering, Shanghai Jiao Tong University, Shanghai, China; ^4^Institute of Medical Robotics, Shanghai Jiao Tong University, Shanghai, China; ^5^National Engineering Research Center of Advanced Magnetic Resonance Technologies for Diagnosis and Therapy (NERC-AMRT), Shanghai Jiao Tong University, Shanghai, China; ^6^Department of Radiology, Ruijin Hospital, Shanghai, China; ^7^Department of Neurosurgery, Brigham and Women's Hospital, Harvard Medical School, Boston, MA, United States

**Keywords:** cerebrovascular disease, neuroimage, evaluation, diagnosis, prediction

Individual variability in cerebrovascular disease (CVD) causes significant variation in diagnosis and treatment. It calls into question standard assessment methods based on clinical trials and creates a harsh reality for prognosis. For example, only one-third of patients with acute ischemic stroke who receive revascularization therapy have a positive outcome, while almost half suffer long-term disability. The limitations of traditional assessment methods are largely responsible for this huge discrepancy in prediction. More exact and personalized evaluation approaches are required. Neuroimaging techniques based on quantitative evaluation are likely to fill this gap as technology develops.

The phrase “time is brain” is especially applicable to acute ischemic stroke (AIS). One of the most difficult issues for neurologists is reducing the time from door to needle. The computerized tomography (CT) scan is the preferred method since it is faster than the magnetic resonance imaging (MRI). However, in exchange for speed, a plain CT scan lacks precision; it can only recognize an ischemic tissue that is at least 24 h post-stroke. Huang et al. conducted a retrospective study in which they introduced a novel CT scan: detector-based spectral CT (SDCT), which recognized ischemia tissue with 96.5% sensitivity and 82.5% specificity within 24 h of AIS. They demonstrated a case in which a scan was performed 6 h after the commencement of the stroke, and SDCT revealed an ischemic area. Because this technology enhanced the accuracy while maintaining the speed of a standard CT, SDCT may become a better choice for AIS diagnosis.

Given the high rate of impairment following AIS, prevention is particularly critical. AIS prevention comprises treating transient ischemic attacks (TIAs) in a timely and effective manner. TIAs, being reversible cerebral ischemia episodes, are difficult to identify using standard imaging methods. Approximately 60% of TIAs do not exhibit positive diffusion-weighted imaging (DWI) signals. Zhou et al. attempted to address this issue by using a novel MRI sequence called diffusion kurtosis imaging (DKI). In this study, 31.6% of DWI-negative TIA patients had positive DKI findings; the DKI-positive sites were highly consistent with symptoms, and recurrent stroke occurred more frequently in the DKI-positive group. DKI has a high value in the diagnosis of TIAs and the prediction of TIA-related strokes, which is beneficial for TIA therapy and AIS prevention.

The most common cause of AIS is cerebral atherosclerosis. Abnormal local vascular or systemic circulation status also has a significant impact on the onset, progression, and prognosis of CVD. Cerebral small vessel disease (CSVD) is a common asymptomatic vascular disorder in the brain. It is frequently missed by patients and overlooked by neurologists. CSVD, on the other hand, can result in symptomatic CVDs such as lacunar stroke, cerebral hemorrhage, and dementia. Numerous works of research have used structural imaging to examine CSVD, but the micro-vessel function of CSVD is still unknown. Fu et al. assessed the hemodynamics of CSVD using transcranial Doppler (TCD)-based critical closing pressure (CrCP). They discovered that CrCP was an independent predictor of CSVD load and was age-related. TCD is ideal for screening population health. Following up on the CSVD burden in the community is useful for determining the link between CSVD burden and symptomatic CVDs.

In addition to CSVD, arteriovenous malformation (AVM) is a local cerebral vascular abnormality. The diagnosis of dural arteriovenous fistula (DAVF), which accounts for 10–15% of AVMs, is difficult. The main reasons are heterogeneous symptoms and a lack of good diagnostic tools. Surgical procedures, such as digital subtraction angiography (DSA), are still used for diagnosis. Although non-invasive radiological procedures like CT and MR angiography are widely employed, their sensitivity and specificity are insufficient to compete with the gold standard. To analyze dural arteriovenous fistula (DAVF), Chen et al. used magnetic resonance angiography (MRA)-based differential subsampling with cartesian ordering (DISCO) sequencing. DISCO demonstrated superior sensitivity, specificity, and accuracy in detecting DAVF with or without cerebral venous thrombosis compared to time of fight (TOF) sequencing. DISCO's performance in DAVF diagnosis is promising.

In contrast to local vascular abnormalities, systemic circulation condition frequently impacts whole-brain perfusion. Cui et al. chose acute leukemia (AL) as their study object. An axial T1 weight and intravoxel incoherent motion (IVIM) sequence were used in their prospective investigation. Gray and white matter perfusion in AL patients and healthy volunteers were compared. In the brain parenchyma of AL patients and healthy volunteers, there was a difference in IVIM values. Although the significance of perfusion irregularities in brain parenchyma in AL is yet unknown, this work offers a new perspective on the pathological process of AL, which will provide new evidence in the diagnosis, evaluation, and treatment of AL and its repercussions.

Multiple dimensions of evaluating CVDs have become a reality as improved neuroimaging techniques continue to revolutionize. Neurologists and radiologists can now use these tools to learn more about specific patients. The difficulty today is figuring out how to handle varied neuroimaging data and extract its usefulness. Nonetheless, the era of personalized and precise treatment is rapidly approaching. With the advancement of neuroimaging techniques, it is envisaged that the pain caused by CVDs would be greatly reduced in the coming era.

## Author contributions

WJ drafted and edited the manuscript. WJ, BL, YF, and YT edited the manuscript. All authors contributed to the manuscript and approved it for publication.

